# Plasmonic and surface-enhanced Raman nanobiosensors for quantitative molecular detection

**DOI:** 10.1186/s11671-026-04697-1

**Published:** 2026-06-02

**Authors:** Yeongbeom Kim, Jaewon Choi, Subin Lee, Yerim Kim, Jisu Park, Kisung Lee, Eunsoo Cho, Jaewon Lee, Kwang Suk Lim, Hyun-Ouk Kim

**Affiliations:** 1 Division of Chemical Engineering and Bioengineering, College of Art, Culture and Engineering, Kangwon National University, Chuncheon, Gangwon State 24341 Republic of Korea; 2 Department of Smart Health Science and Technology, College of Engineering, Kangwon National University, Chuncheon, Gangwon State 24341 Republic of Korea; 3 Institute of Fermentation of Brewing, Kangwon National University, Chuncheon, 24341 Republic of Korea; 4 Institute of Industrial Technology, Kangwon National University, Chuncheon, 24341 Republic of Korea; 5 Department of Mechanical and Aerospace Engineering, University of Missouri, Columbia, 65211 USA; 6 MU Materials Science and Engineering Institute, University of Missouri, Columbia, MO 65211 USA; 7 Department of Chemical and Biomedical Engineering, University of Missouri, Columbia, 65211 USA

## Abstract

Plasmonic surface-enhanced Raman scattering (SERS) nanobiosensors employ nanoscale electromagnetic field amplification to achieve ultrasensitive, multiplex molecular detection. This review systematically outlines the fundamental plasmonic principles, nanostructure engineering strategies, and surface chemical functionalization approaches that dictate sensor performance. Quantitative analysis methodologies—including internal standards, ratio-based quantification, and machine learning-driven spectral interpretation—are critically examined. Potential clinical and field applications are highlighted through examples involving nucleic acids, proteins, pathogens, and environmental toxicants. Key technical challenges, such as reproducibility, scalable manufacturing, and methodological standardization, are discussed in detail. Finally, future directions are proposed, emphasizing single-molecule quantification, in vivo SERS applications, and the integration of sustainable materials into sensor design.

## Introduction

Plasmonic nanobiosensors employ localized surface plasmon resonance (LSPR) generated by metallic nanostructures to transduce molecular binding events and concentration variations into measurable optical signals [1, 2]. Compared with conventional fluorescence-based techniques, these sensors demonstrate superior resistance to photobleaching and provide rich spectral information on molecular interactions [3, 4]. Surface-enhanced Raman scattering (SERS) further amplifies plasmonic effects, enabling the detection of molecular vibrational features as intense signals from minute samples, with sensitivity approaching the single-molecule level [4–6]. Consequently, SERS has emerged as a promising sensing technology for precise biomarker detection.

The growing emphasis on precision medicine has heightened the demand for quantitative monitoring of concentration and conformational changes, extending beyond simple molecular identification [7]. Applications such as early disease diagnosis, therapeutic response evaluation, and environmental contamination assessment increasingly require reliable quantitative analysis [8, 9]. In this context, the utility of plasmonic and SERS nanobiosensors depends not only on their ultrahigh sensitivity but also on their reproducibility and analytical robustness [10]. However, SERS signals are often non-uniformly enhanced due to variations in nanostructure morphology and spatial distribution, leading to inter-assay variability [11]. Addressing these challenges necessitates precise control of surface architecture, incorporation of internal reference standards, and the application of spectral correction algorithms and database-driven approaches [12, 13].

**Fig. 1:**
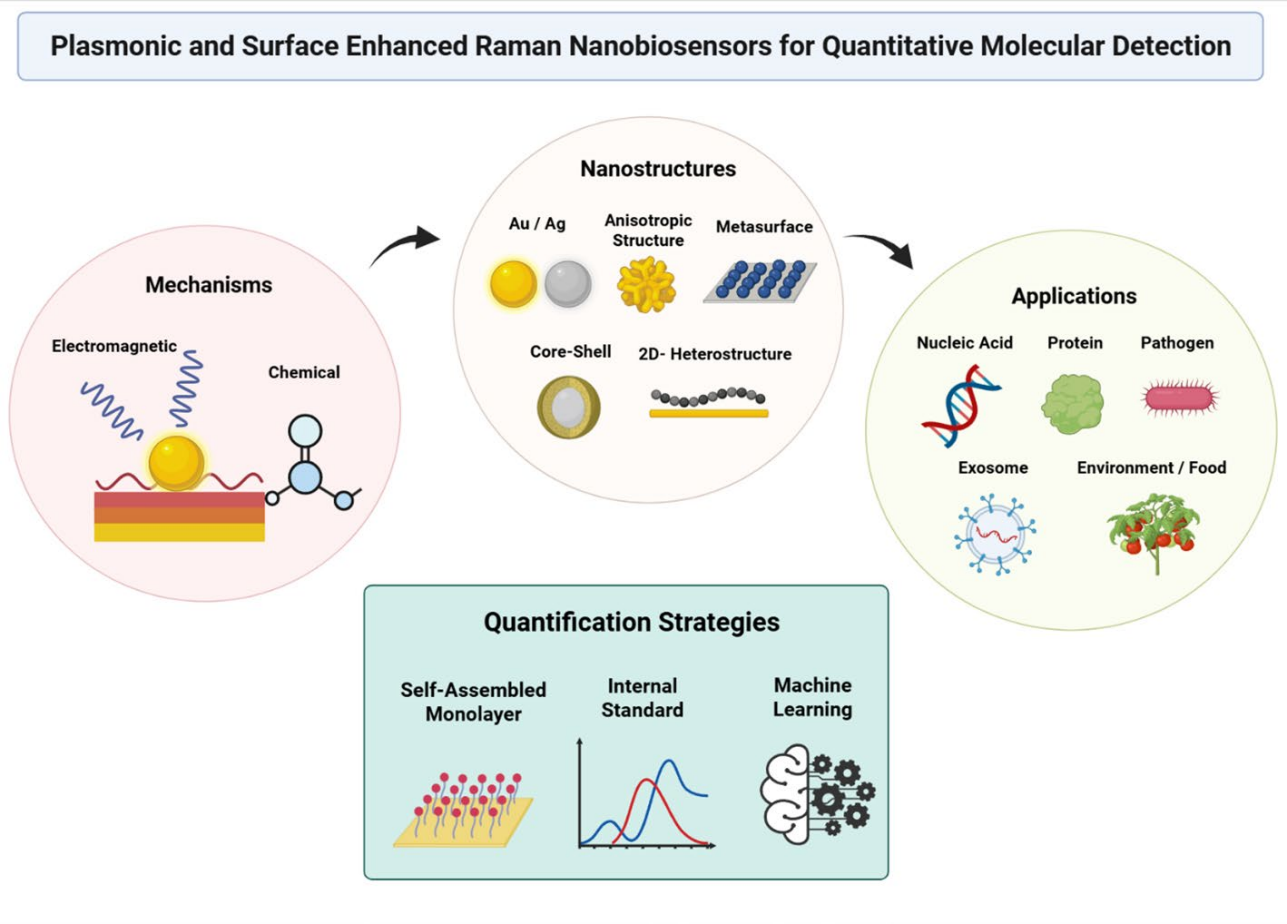
Framework of plasmonic enhancement mechanisms for quantitative Surface-Enhanced Raman Scattering (SERS) biosensing

This review responds to these technological needs by summarizing current progress and future perspectives. We comprehensively examine fundamental design principles, performance metrics, surface chemistry optimization strategies, data-processing methodologies, and representative application cases. Together, these advances highlight the potential of plasmonic and SERS nanobiosensors as reliable platforms for quantitative molecular analysis. To provide an overview of these interconnected themes, Fig. 1 presents a consolidated schematic illustrating the fundamental mechanisms, representative nanostructures, key application areas, and emerging quantification strategies discussed in this review.

This schematic illustrates the mechanisms underlying quantitative SERS biosensing. Localized surface plasmon resonance (LSPR)–driven electromagnetic hotspots and surface chemical interactions synergistically amplify Raman signals, while controlled nanostructure geometry, tailored surface functionalization, and advanced data-processing strategies ensure signal uniformity and quantitative reliability. Created with BioRender.com.

## Fundamentals of plasmonic and surface-enhanced Raman scattering

### Electromagnetic enhancement vs. chemical enhancement mechanism

Electromagnetic enhancement occurs when the strong local electric field generated by the LSPR of a metallic nanostructure interacts with the induced molecular dipole, thereby amplifying Raman polarization [14]. The resulting signal scales with the fourth power of the local field intensity and is governed by factors such as the interparticle gap, tip curvature, nanostructure geometry, optical constants of the metal and dielectric, and the spectral overlap between the excitation wavelength and the resonance band [15, 16]. This enhancement pathway is quantitatively described by the electromagnetic factor G_EM in Eq. (2), which reflects the local-field contributions at both the incident and scattered frequencies [17].

In contrast, chemical enhancement originates from charge-transfer states formed when molecules adsorb on the metal surface [18]. These states alter the polarizability tensor, selectively enhance specific vibrational modes, and operate over angstrom-scale distances [19]. As indicated in Eq. (3), the resulting chemical enhancement factor G_Chemtypically contributes one to two orders of magnitude, depending on the strength of metal–molecule electronic coupling [20].

The overall SERS intensity is ultimately determined by the multiplicative interplay of these two mechanisms, as summarized in Eq. (1) [21]. Because both G_EM and G_Chem are highly sensitive to nanostructure morphology, surface chemistry, and molecular binding configuration, uncontrolled variations can lead to significant signal fluctuations and reduced quantitative reliability [22]. Ensuring geometric uniformity and consistent surface functionalization is therefore essential for obtaining reproducible and concentration-dependent responses in practical biosensing environments [23]. This mechanistic framework provides the physical basis for the quantification strategies discussed in subsequent sections [24].

(1) Total SERS enhancement:

$${G}_{SERS}={G}_{EM}\times {G}_{Chem}$$

(2) Electromagnetic enhancement factor (${G}_{EM}$):

$${G}_{EM}={\mid \frac{{E}_{local}({\omega}_{in})}{{E}_{0}({\omega}_{in})}\mid }^{2}\times {\mid \frac{{E}_{local}({\omega}_{out})}{{E}_{0}({\omega}_{out})}\mid }^{2}$$

(3) Chemical enhancement factor (${G}_{Chem}$):

$${G}_{Chem}\approx {10}^{1}-{10}^{2}$$

### Hotspot formation, signal uniformity, and statistical characteristics

Hotspots arise from the coupling of localized surface plasmons at interparticle gaps, sharp tip curvatures, and particle–substrate junctions [25]. Gap-mode resonance and the lightning-rod effect concentrate the electromagnetic field, while resonance conditions are determined by excitation wavelength, polarization, and the surrounding refractive index [26].

Intensity uniformity is highly sensitive to variations in gap size, periodic arrangement, surface roughness, and scattering losses [27]. Achieving quantitative reliability requires minimizing spatial variation through deterministic gap control, periodic array design, large-area uniform fabrication, and surface passivation [28].

Signal distributions are typically dominated by a small number of intense hotspots and often follow log-normal statistics [21]. Variability is further influenced by molecular occupancy and adsorption–desorption kinetics, which can cause single-point measurements to overestimate mean intensity [29]. Mapping-based spatial and temporal averaging, combined with internal standards, can reduce variance and improve reproducibility [30].

Key quantitative metrics include hotspot density, effective area fraction, field coefficient of variation, and interpixel correlation length [31]. Aggregate-based substrates provide high hotspot density but suffer from poor uniformity, whereas lithography-based substrates yield lower density but greater reproducibility [32, 33]. The optimal substrate choice depends on balancing sensitivity requirements, surface chemistry compatibility, and analysis time [34].

Although hotspots enable ultrahigh sensitivity, they remain vulnerable to structural variations that compromise reproducibility in quantitative analysis [35]. Therefore, stable quantitation in real-world environments requires systematic management of hotspot statistical characteristics and careful control of structural variability [36]. This perspective emphasizes the importance of designing SERS platforms with controllable architectures rather than relying solely on high-intensity structures [37].

### Raman cross sections, selection rules, and reaction rate considerations

The Raman scattering cross-section is determined by the derivative of a molecule’s polarizability tensor with respect to its vibrational coordinates and increases markedly when aligned with an electronic resonance [38]. In SERS, both electromagnetic and chemical enhancements contribute to the effective scattering cross-section, with the relative contribution of each vibrational mode depending on molecular orientation and the direction of the local electric field at the surface [39].

Although selection rules are dictated by molecular symmetry, adsorption onto a metallic surface disrupts this symmetry, preferentially enhancing polar components parallel to the metal–molecule bond axis [40]. These modified “surface selection rules” are strongly influenced by adsorption geometry, orientation distribution, and surface coverage, which collectively determine mode contrast and polarization dependence [41].

From a kinetic perspective, analyte diffusion, adsorption–desorption dynamics, competitive binding, and surface-induced chemical changes govern SERS signal formation. Under isothermal stopped-flow conditions, the response follows a Langmuir series of rate equations and equilibrium constants, with binding affinity and hotspot accessibility defining both the kinetic detection limit and the linearity of the response [42]. Laser power, focusing, and thermal effects further modulate the scattering cross-section and selection rules by inducing local heating and altering solvent or electrolyte composition [43].

Because Raman cross-sections and selection rules are highly sensitive to molecular orientation, binding state, and adsorption kinetics, structural uncertainties often lead to spectral variability even at identical analyte concentrations [44]. These factors impose significant constraints on maintaining concentration-dependent signals in quantitative SERS analysis [45]. Thus, functionalization strategies that control binding geometry and adsorption kinetics are essential to improving reproducibility [46]. Ultimately, elucidating the physicochemical mechanisms of molecule–surface interactions provides a critical foundation for advancing quantitative SERS platforms.

## Nanostructured materials and architectures

### Colloidal nanoparticles and anisotropic structures (Au, Ag, Alloys, etc.)

Colloidal nanoparticles are nanometer-scale metal particles dispersed stably in solution, typically stabilized by ligands or surfactants to prevent aggregation. They exhibit LSPR that depends on particle size and the surrounding refractive index, offering straightforward synthesis, versatile functionalization, and tunable optical properties [47, 48].

Colloidal nanoparticles can be isotropic (e.g., nanospheres) or anisotropic, with unequal dimensions along specific axes (e.g., nanorods, nanoplates, nanoprisms, nanostars). Anisotropic structures feature sharp tips and corners, display longitudinal and transverse plasmon mode separation, and enable localized field enhancement and wavelength tunability, thereby providing greater design flexibility for SERS sensing [49, 50].

**Fig. 2:**
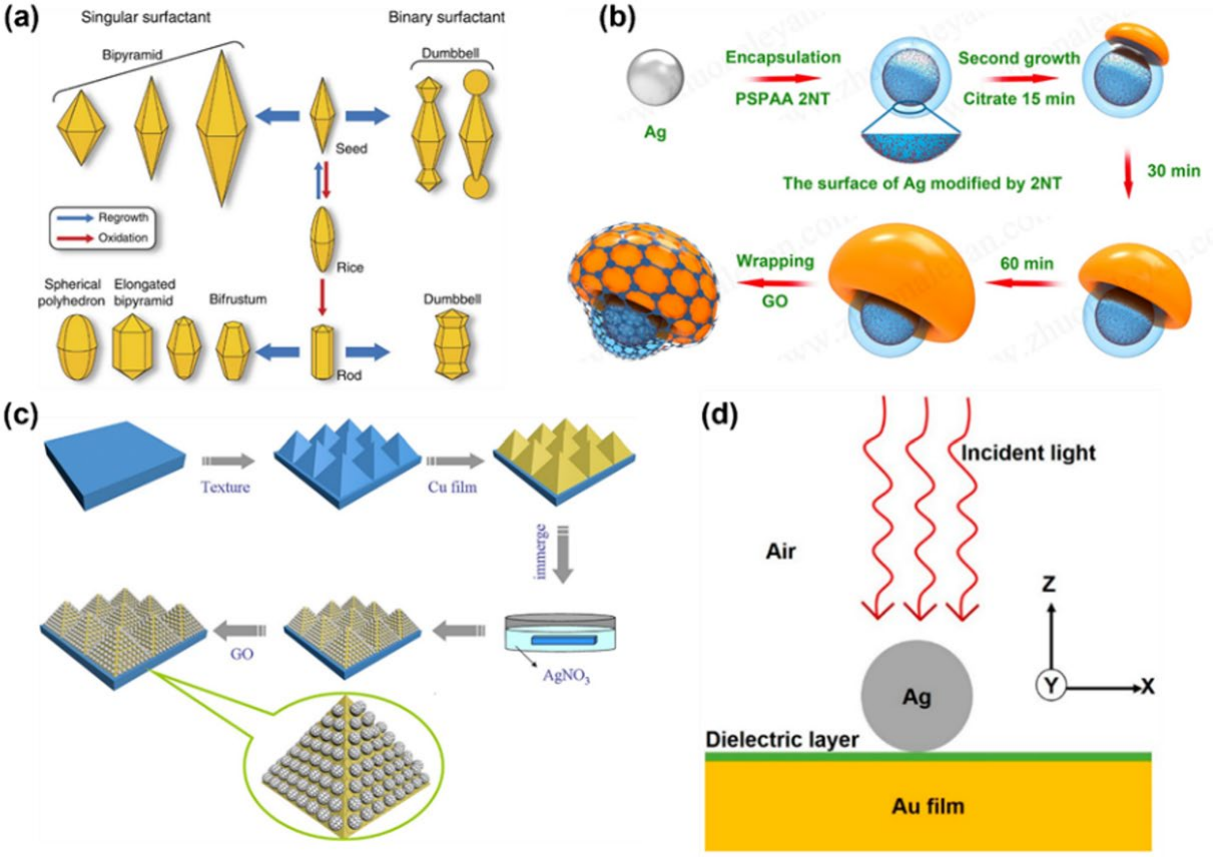
Representative nanostructured and hybrid platform designs for plasmon-enhanced SERS applications. **a** Schematic illustration of shape-controlled anisotropic gold nanocrystals derived from seed-mediated regrowth and oxidation processes, showing the evolution of bipyramidal and related geometries under different surfactant conditions. Reproduced from Lee et al. [52], CC BY 4.0. **b** Schematic representation of the stepwise synthesis of a graphene oxide-wrapped silver nanomushroom structure, including surface modification, secondary growth, and final wrapping with graphene oxide. Reproduced from Jiang et al. [62], CC BY 4.0. **c** Fabrication process of a hierarchical graphene oxide/Ag nanoparticle/Cu film@pyramidal Si substrate, highlighting sequential texturing, metal deposition, immersion-based nanoparticle formation, and graphene oxide integration. Reproduced from Li et al. [64], CC BY 4.0. **d** Schematic model of a nanoparticle-on-film plasmonic architecture composed of a silver nanoparticle, dielectric spacer layer, and gold film under incident light excitation, illustrating a metal–dielectric hybrid configuration for localized field enhancement. Reproduced from Devaraj et al. [65], CC BY 4.0

These nanostructures are most commonly composed of Au or Ag. Au–Ag alloys and core–shell configurations combine the advantages of both metals, balancing resonance wavelength, stability, and enhancement performance [51]. By co-engineering metal composition and structural anisotropy, it is possible to achieve both high sensitivity and tailored spectral characteristics. Representative plasmonic nanostructures and anisotropic colloidal geometries are illustrated in Fig. 2a, demonstrating how particle size, aspect ratio, and tip curvature govern LSPR behavior and electromagnetic field confinement [52]. While colloidal and anisotropic nanostructures provide high enhancement efficiencies, they remain limited by reproducibility: even minor variations in particle morphology or surface condition can significantly affect signal consistency [53]. Therefore, standardized manufacturing processes that minimize batch-to-batch variation and ensure consistent functionalization are essential for quantitative SERS analysis [54]. Such controllability in structural design is a critical requirement for achieving both ultrahigh sensitivity and reliable quantitation.

### Nanopatterning and lithography-based substrates (metasurfaces, arrays)

Nanopatterning and lithography are fabrication techniques that enable deterministic arraying of subwavelength structures and simultaneous engineering of LSPR and lattice effects [55]. Metasurfaces modulate the effective refractive index and phase response through the shape, height, period, and duty cycle of nanoantennas, while gap–plasmon structures such as bowties, nanogap antennas, and metal–dielectric–metal resonators generate stable and intense near fields [56].

Periodic arrays of nanodisks, nanofillers, or nanoholes produce narrowband resonances that couple LSPR with surface lattice resonance (SLR), ensuring selective enhancement through polarization control, incident angle tuning, wavelength matching, and spatial field uniformity [57]. Electron-beam lithography enables ultrafine gaps and complex geometries with high precision, whereas nanoimprinting, interference lithography, and stepper processes provide large-area replication and wafer-scale reproducibility [58].

Lithographic substrates allow precise control of hotspot location and density, reducing statistical signal variance and facilitating integration with internal standards and multiplexed Raman tags [59]. They are also compatible with microfluidic channels, on-chip heating modules, and wavefront control systems [60]. Typical materials include Au or Ag thin films, adhesive layers, and dielectric intercalation layers, which enhance oxidation stability, suppress nonspecific adsorption, and improve long-term consistency and reusability [61].

Figure 2b illustrates lithographically patterned plasmonic arrays and metasurfaces, highlighting deterministic control of gap spacing, periodicity, and resonance coupling [62]. While lithography-based substrates provide high structural accuracy and are well-suited for quantitative analysis, their process characteristics present a duality: scalability and accessibility are often limited by high fabrication costs and equipment variability [63]. Consequently, ensuring consistency across the entire manufacturing ecosystem—not only structural precision—is essential for the practical deployment of quantitative SERS platforms.

### Hybrid and tunable platforms (core–shell, metal–dielectric, two-dimensional heterogeneous structures)

Hybrid platforms integrate multiple materials or resonant modes to overcome the physical limitations of single-metal structures and establish multilayered enhancement mechanisms [66]. Metal–metal core–shell architectures enable precise tuning of resonant wavelengths and field distributions through plasmonic coupling between the core and shell layers. Alloyed or porous shells have been explored to stabilize resonance properties while simultaneously expanding hotspot regions [67].

Metal–dielectric architectures combine the strong electromagnetic response of metals with the long-range confinement of dielectrics, thereby enhancing near-field localization while reducing energy loss [68]. Metal–dielectric–metal resonators and nanoparticle-on-mirror configurations are being actively developed to tune spectral linewidths and control hotspot positioning [69].

Two-dimensional material–based heterostructures are also under intensive investigation. Graphene and transition metal chalcogenides provide charge-transfer pathways and atomic-scale distance control, complementing chemical enhancement while suppressing metal-surface quenching [70]. Monolayers are engineered to optimize metal–molecule interaction distances, improving reproducibility and selectivity.

These hybrid structures offer tunable design parameters—including composition, layer thickness, and interfacial properties—that enable simultaneous control over resonance behavior, stability, and selectivity [71]. Figure 2c illustrates representative hybrid and tunable architectures, including metal–dielectric and two-dimensional heterogeneous interfaces that synergistically combine electromagnetic and chemical enhancement mechanisms [64]. Table 1 summarizes representative plasmonic nanostructures, materials, gap sizes, and experimentally reported enhancement factors, providing a concise overview of how enhancement efficiency and signal uniformity vary across structural designs.

**Table 1:** Representative plasmonic architectures, materials, and experimentally reported enhancement factors (EFs) in SERS studies

Structure	Material	Gap size (nm)	Enhancement factor (EF)	Reference
Nanoparticle dimer	Au, Ag	1–3	10^8^–10^10^	[72]
Nanoparticle-on-mirror (NPoM)	Au@SiO_2_	1–2	10^9^–10^11^	[73]
3D hydrogel plasmonic network	Au nanorods/AgNP in hydrogel	5–10	10^7^–10^9^	[74]
Lithographic metasurface/nanoarray	Ag nanoarray	10–20	10^6^–10^7^	[75]
Hierarchical nanowire assembly	Ag–Cu hybrid	variable	10^7^–10^9^	[76]

While hybrid platforms provide functional advantages by leveraging multiple resonance modes and interface effects, their complexity significantly broadens the scope of signal interpretation. In systems where electromagnetic and chemical contributions overlap, disentangling individual factors becomes challenging, leading to uncertainty in quantitative analysis. Consequently, although these platforms excel in achieving high sensitivity, quantitative reliability depends strongly on interface design and precise control of material properties.

## Surface chemistry and biofunctionalization

### Self-assembled monolayers, biomolecular binding, and directional control

Self-assembled monolayers (SAMs) are molecular films formed by the spontaneous alignment of binding groups such as thiols or silanes on metal or oxide surfaces, providing uniform surface functionalities for subsequent modification. The attachment of biorecognition molecules—including antibodies, nucleic acids, or peptides—enhances target selectivity. Binding strategies are generally classified as covalent bonding, click chemistry, or affinity-based interactions (e.g., streptavidin–biotin) [77–79].

The orientation of recognition molecules is critical: depending on how the active site is exposed or shielded, binding efficiency and specificity can vary substantially. Thus, directional control plays a central role in optimizing functionalization. Adjusting the metal–molecule distance through spacer molecules or molecular length control minimizes electronic quenching and improves both the linearity and modal distinguishability of Raman signals [80, 81].

**Fig. 3:**
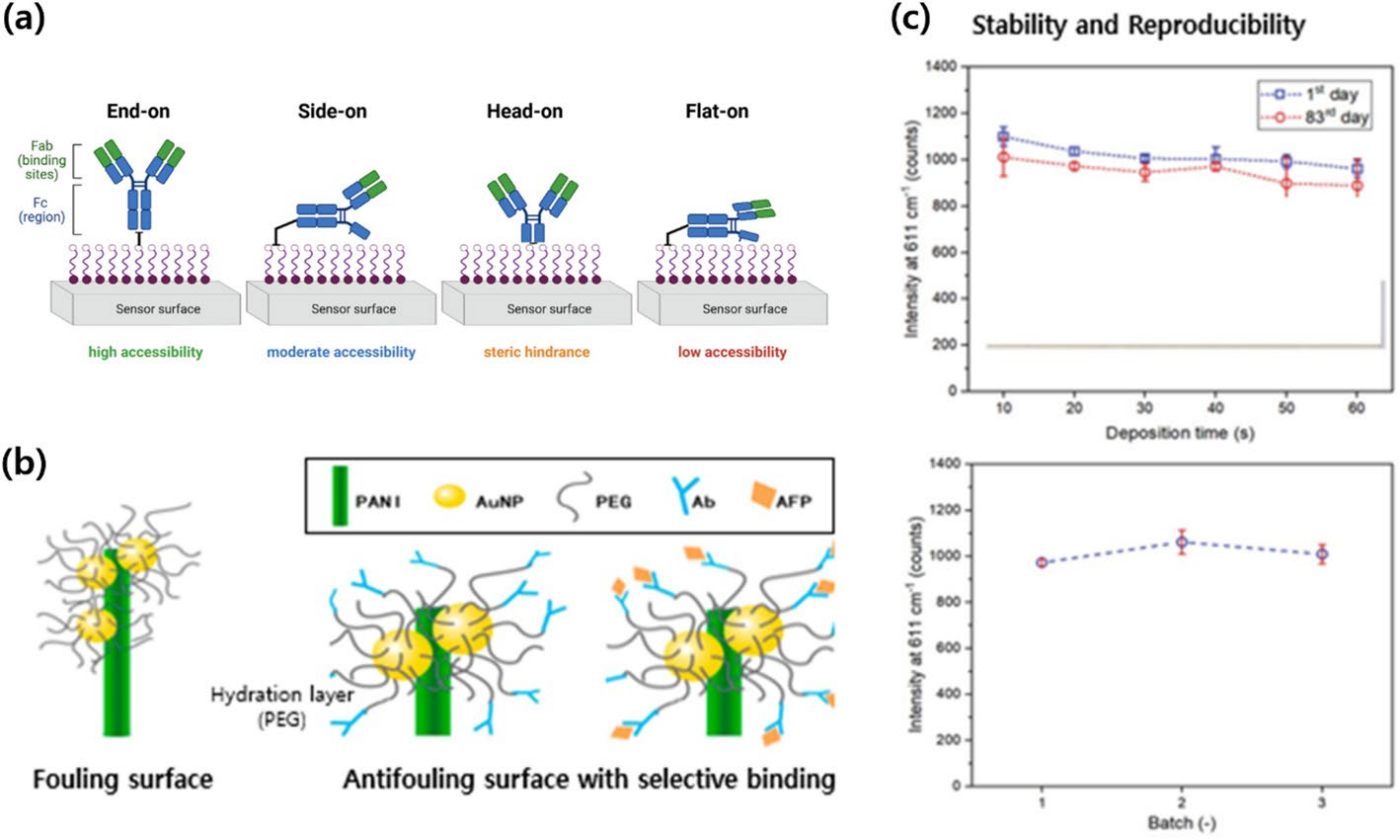
Surface chemistry strategies for quantitative SERS biosensing. **a** Schematic illustration of self-assembled monolayer (SAM)-based surface functionalization enabling controlled antibody orientation on metal substrates, demonstrating end-on, side-on, head-on, and flat-on configurations and their influence on antigen-binding site exposure. Schematic concept inspired by ref. [82], redrawn using BioRender.com. **b** Schematic representation of antifouling surface modification using polymer brush layers, illustrating the formation of a hydration barrier that suppresses nonspecific adsorption while maintaining selective analyte accessibility to plasmonic hotspots. Reproduced from Song et al. [84], CC BY 4.0. **c** Demonstration of stability and reproducibility of SERS substrates, including signal retention over time and consistent signal intensity across independent fabrication batches, indicating reliable quantitative performance. Reproduced from Li et al. [25], CC BY 4.0

These functionalization strategies enhance the quantitative performance of SERS sensing by guiding analytes toward hotspots in favorable orientations, reducing nonspecific interactions, and minimizing measurement-to-measurement variability. Figure 3a illustrates SAMs enabling controlled biomolecular orientation and functional anchoring on metal surfaces. SAM-based functionalization provides a structural foundation for quantitative analysis, ensuring precise control of biomolecule orientation and accessibility [82].

However, if SAM integrity or density is not maintained uniformly across a real surface, analyte binding geometry can vary significantly, introducing errors in quantitative analysis. Therefore, quantitatively understanding how subtle variations in SAM formation influence signal reliability remains a key challenge in the design of robust surface functionalization strategies [83].

### Suppressing nonspecific adsorption, mass transport, and improving analyte accessibility

Quantitative SERS analysis requires careful control of the surface environment to prevent nontarget molecules from adsorbing onto metal surfaces, as such interactions can distort signals and hinder analyte access. Strategies such as polyethylene glycol passivation, charge-blocking layers, and protein-based blockers are commonly employed to suppress nonspecific adsorption of molecules with hydrophobic or electrostatic residues [85, 86].

Because hotspots—the regions of strongest SERS enhancement—are highly localized, efficient mass transport is critical. Approaches such as shortening diffusion distances, controlling microfluidic flow, and introducing porous architectures facilitate effective analyte migration to these regions. Physicochemical parameters, including molecular size, charge, and ionic strength, directly influence hotspot accessibility; restricted access can lead to signal saturation or large statistical fluctuations, even in highly sensitive substrates [87, 88].

Therefore, minimizing nonspecific adsorption and tailoring both the surface and fluidic environment to ensure proper analyte delivery to active sites are essential design considerations for achieving signal linearity, spatial uniformity, and repeatability while maintaining concentration dependence. Figure 3b illustrates antifouling polymer layers that prevent nonspecific adsorption while maintaining analyte accessibility to SERS hotspots [89].

Nonspecific adsorption and mass transfer limitations are key factors that define the upper limit of quantification, driven more by the experimental environment than by signal interpretation. In complex matrices such as biological samples, these nontarget interactions can amplify spectral variability. Consequently, surface passivation and mass transfer design serve as practical control variables that determine the stability of quantitative analysis, rather than directly enhancing SERS sensitivity [90, 91].

### Stability, storage, and inter-product consistency (reproducibility)

Robust SERS performance in practical applications depends on coordinated control of the entire analytical workflow, not only on the structural uniformity of the substrate [92–94]. Even when plasmonic nanostructures are fabricated with similar morphology and retain acceptable stability during storage, signal variation may still arise from differences introduced during sample preparation. Parameters such as analyte concentration, solvent composition, incubation time, droplet deposition, drying pattern, and matrix complexity can all influence molecular adsorption and hotspot accessibility, thereby affecting both signal intensity and spectral profile. These effects become even more important for biological and environmental samples, where proteins, salts, and other coexisting species may interfere with target localization or alter local surface interactions [93]. Figure 3c illustrates the stability and batch reproducibility of SERS substrates, demonstrating consistent signal retention during storage and reuse. However, this substrate level result should be interpreted as one important part of reproducibility, rather than as a complete explanation of measurement consistency.

Equally important are instrumental and environmental factors that influence spectral comparability across measurements. Small variations in laser power, focus position, wavelength calibration, detector sensitivity, acquisition conditions, and spectral preprocessing can produce measurable differences even when the same substrate platform is used. In addition, temperature, humidity, ambient contamination, and operator dependent handling may further affect performance during storage and analysis. Previous studies have therefore highlighted the need for standardized sample preparation procedures, routine instrument calibration, controlled measurement environments, and systematic quality assessment across fabrication batches and measurement sessions [93, 94]. The use of internal standards, reference analytes, automated fabrication methods, and statistical validation of batch to batch consistency has also been proposed as practical strategies to strengthen robustness. Taken together, these considerations show that reliable reproducibility in SERS is governed by integrated control of substrate design, sample handling, instrumental conditions, and environmental management.

## Quantitative assay design, calibration, and data analytics

### Internal standards, ratiometric, and isotope-encoded approaches

Absolute signal intensity in quantitative SERS biosensing is often unstable because it depends on multiple variables, including nanostructure morphology, surface chemistry, and measurement conditions [95]. To address this variability, internal standards are introduced to correct sample-to-sample fluctuations. In the ratiometric approach, quantification is achieved by calculating the intensity ratio (I_analyte_/I_standard_) between a characteristic Raman band of the analyte (e.g., the G band at 1580 cm⁻^1^) and a reference peak of the internal standard (e.g., 1650 cm⁻^1^). This compensates for instrument drift, optical path deviations, and inefficiencies in signal transduction [96].

The isotope encoded strategy replaces part of the analyte with isotopes such as deuterium, carbon 13, or nitrogen 15, producing a predictable shift in selected Raman bands [97–99]. Because isotope labeled molecules usually preserve the key chemical structure and target recognition characteristics of their unlabeled counterparts, they can act as closely matched internal references for signal normalization and multiplex target tracing [97]. This feature can improve measurement consistency and reduce variability arising from instrumental fluctuation, signal drift, and substrate heterogeneity.

However, in complex biological matrices, isotope encoding does not always lead to true absolute quantification. Matrix components such as proteins, lipids, salts, and other endogenous molecules can compete for surface adsorption, alter hotspot accessibility, and generate overlapping background signals that distort peak ratios. In addition, labeled and unlabeled species may not undergo exactly the same extraction, enrichment, transport, or local surface binding processes, which can leave residual matrix dependent bias uncorrected. Therefore, isotope encoding is more appropriately described as a strong strategy for improving quantitative reliability rather than a universal solution for absolute quantification.

**Fig. 4:**
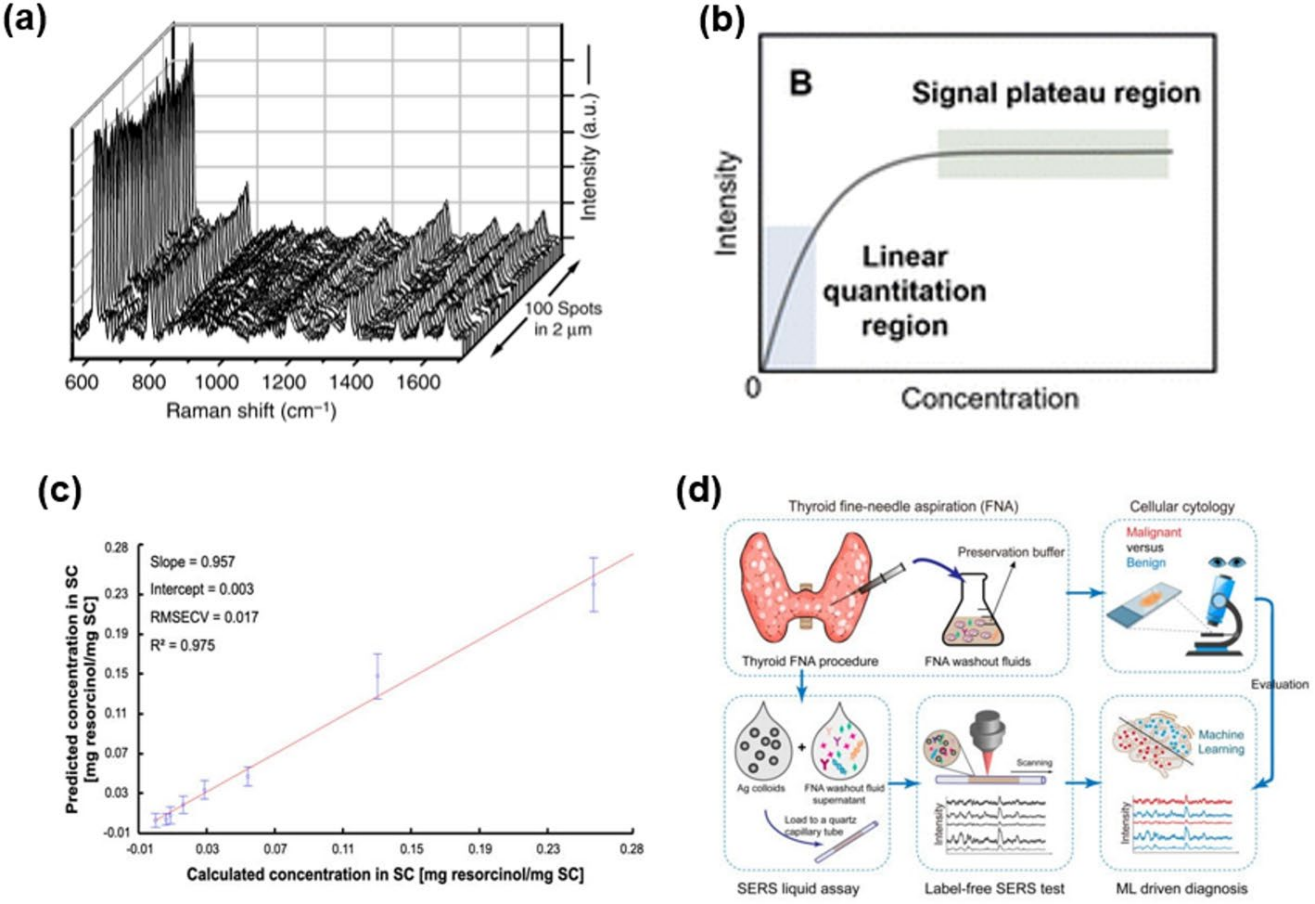
Integrated strategies for quantitative SERS biosensing and data analysis. **a** Representative SERS spectra acquired from multiple hotspots, illustrating significant signal variability and challenges in quantitative reproducibility. Reproduced from Cong et al. [100], CC BY 4.0. **b** Conceptual calibration model showing the linear quantification region and signal saturation at high analyte concentrations. Reproduced from Xu et al. [101], CC BY 3.0. **c** Multivariate regression (e.g., PLSR) demonstrates strong correlation between predicted and measured concentrations, enabling accurate quantification. Reproduced from Kichou et al. [102], CC BY 4.0. **d** Machine learning–assisted SERS workflow for automated spectral analysis and diagnostic prediction. Reproduced from Gao et al. [103], CC BY 4.0

Overall, these strategies fundamentally enhance the reliability and clinical applicability of SERS-based biomarker detection. Figure 4a illustrates an internal-standard-assisted ratiometric quantification scheme in which a reference molecule is co-measured with the analyte to correct intensity fluctuations, thereby improving quantitative accuracy and reproducibility [100].

However, the choice and labeling method of internal standards can increase system complexity. In biological samples, competition between standards and analytes for surface adsorption may introduce new biases. Thus, internal standard-based quantitation functions not only as a calibration technique but also as a quantitative model that requires careful consideration of additional design variables.

### Calibration models and metrology (LOD/LOQ, dynamic range, uncertainty)

Quantification in SERS biosensing relies on calibration curves that relate sensor response (e.g., Raman peak intensity, intensity ratios, or spectral regions) to a series of standard concentrations. While linear regression is the simplest model, many SERS systems are better described by exponential, logistic, or polynomial functions due to nonlinearities arising from Raman scattering efficiency, surface saturation, or concentration-dependent aggregation [22, 104].

The limit of detection (LOD) is typically defined as three times the standard deviation of the background noise (S/N = 3), whereas the limit of quantitation (LOQ) corresponds to the lowest concentration that permits reliable quantification under a stricter criterion (S/N = 10). The dynamic range extends from the LOQ to the upper concentration limit where signal saturation occurs. A broader dynamic range allows a single sensor to accommodate diverse physiological and clinical concentration levels [105, 106].

Measurement uncertainty is expressed as expanded uncertainty (approximately 95% confidence, *k* = 2), incorporating contributions from curve-fitting error, standard material purity, volumetric error, and nanostructural heterogeneity. This uncertainty governs the diagnostic reliability of SERS biosensors and their compliance with environmental and clinical standards. Therefore, rigorous calibration models and adherence to metrological principles are prerequisites for establishing SERS as a reliable quantitative tool [107, 108].

Figure 4b illustrates a Langmuir-type calibration curve, which exhibits nonlinear saturation at high analyte concentrations due to the finite number of enhancement sites. This relationship defines the sensor’s dynamic range and detection limits, providing a framework for quantitative calibration and uncertainty evaluation in SERS biosensing [101]. In highly variable environments such as biological samples, nonlinearity and surface saturation frequently distort the analysis range, underscoring the importance of understanding both the limitations and error structure of the calibration model. Ultimately, adopting a quantitative metrological approach is essential for advancing SERS from a high-sensitivity sensor to a robust diagnostic platform.

### Spectral preprocessing, multivariate regression, and machine learning

SERS spectra often exhibit low signal-to-noise ratios due to fluorescence background, accidental Raman scattering, and instrumental noise. Therefore, spectral preprocessing is essential to remove background signals and normalize spectral intensity, thereby improving the reliability of quantitative analysis. Techniques such as baseline correction, spectral normalization, smoothing, and peak alignment enhance characteristic vibrational features of analytes while reducing system variability [109, 110].

Unlike univariate methods that rely on single peak intensities, multivariate regression utilizes the entire spectral dataset to predict analyte concentrations [111]. Representative models include partial least squares regression (PLSR) and principal component regression (PCR), which effectively mitigate interference from overlapping peaks, background fluctuations, and nanostructural heterogeneity [112].

Machine learning has become a valuable analytical tool in SERS because it can identify complex spectral patterns that are difficult to resolve through conventional peak based analysis alone. Representative applications include multivariate regression models for quantitative prediction and deep learning models for automated spectral classification in complex samples [113–115]. Figure 4c and d illustrate these representative workflows, where preprocessed spectral data are used for concentration prediction through multivariate modeling and for automated pattern recognition through a convolutional neural network [102, 103]. These approaches are especially useful when band overlap, baseline fluctuation, and matrix interference limit the reliability of direct spectral interpretation.

However, these methods still have important limitations. Their performance is often strongly influenced by the size, diversity, and quality of the training dataset, which increases the risk of overfitting when sample numbers are limited, or the data are not sufficiently representative [116, 117]. In addition, highly complex models such as deep neural networks may offer limited interpretability, making it difficult to determine which spectral features primarily drive the prediction outcome. Their broader practical use can also be restricted by poor transferability across different instruments, substrates, and acquisition conditions, because SERS spectra can vary substantially between platforms. Therefore, although Fig. 4c and d demonstrate the analytical potential of machine learning assisted SERS, these methods should be considered supportive tools that require rigorous validation and careful evaluation of generalizability.

## Applications in biosensing

### Nucleic acids and proteins: expression profiling and variant detection

SERS-based biosensors are widely applied for the quantitative detection of nucleic acids and proteins [118]. Nucleic acids (e.g., DNA and RNA) can be translated into distinguishable Raman signals using sequence-specific probes and nanostructured plasmonic surfaces [119]. Single-nucleotide polymorphism (SNP) detection and microRNA (miRNA) expression profiling have been quantified down to the picomolar level, reflecting the high sensitivity of SERS platforms [120, 121].

Protein detection is achieved through immune-based sensors, such as antibody-conjugated SERS probes, which enable direct quantification of specific biomarkers. In addition, biological abnormalities—including protein conformational changes, post-translational modifications, and overexpression—can be analyzed with high precision [122]. Representative clinical applications include quantification of prostate-specific antigen (PSA) in serum using a gold nanoparticle-based SERS sensor, which achieved an LOD of 0.5 ng mL^−1^ and a linear dynamic range of 1–100 ng mL^−1^, clearly distinguishing the clinical reference value of 4 ng mL^−1^ [123]. Similarly, SERS quantification of plasma amyloid-β (Aβ_42_) peptide for Alzheimer’s disease diagnosis yielded an LOD of 10 pg mL^−1^ and a quantification range of 50 pg mL^−1^–10 ng mL^−1^ using a sandwich immunoassay configuration, demonstrating statistically significant differences (*p* < 0.01) between patient and control groups [124]. Likewise, quantification of miRNA-21, a cancer-associated biomarker, achieved an LOD of 1 fM and a six-order dynamic range (10 fM–10 nM) using an isotope-labeled internal standard, revealing an average 8.5-fold higher expression level in serum from breast cancer patients compared with healthy controls [125].

These quantitative SERS detection capabilities demonstrate high accuracy and reliability across diverse biomedical applications, including clinical diagnostics, therapeutic monitoring, and drug response evaluation. However, nucleic acid and protein analysis can complicate signal interpretation due to structural diversity and variations in surface binding states. The molecular-level structural sensitivity offered by SERS directly contributes to quantitative accuracy, but competitive binding and nonspecific adsorption in biological matrices introduce variability. Therefore, the selectivity of molecular recognition elements and the precision of surface design are critical determinants of practical performance in clinical applications. A SERS platform optimized for each biomarker thus forms a key foundation for diagnostic accuracy.

### Pathogens, exosomes, and circulating biomarkers: clinical and point-of-care use

For rapid diagnosis and monitoring of infectious diseases, SERS biosensors have been implemented as nanostructure-based immunosensors capable of recognizing pathogens—including viruses, bacteria, fungi—along with their protein antigens, nucleic acids, and characteristic metabolites [126]. Quantification has been achieved with an LOD of 0.08 pg mL^−1^ and a dynamic range of 0.1–100 pg mL^−1^, yielding sensitivity and specificity greater than 95% compared with RT-PCR [127].

Exosomes, nanosized vesicles (50–150 nm) secreted by cells, serve as circulating biomarkers carrying disease-related proteins and nucleic acids. They can be directly isolated from biofluids such as blood, urine, and saliva, and subsequently SERS-encoded with surface protein markers (e.g., PD-L1, EpCAM) and internal nucleic acids. Diagnostic exosome quantification achieved an LOD of 50 exosomes μL^−1^ and a linear dynamic range spanning five orders of magnitude (50–500,000 exosomes μL^−1^), revealing an average 12-fold higher concentration in cancer patient serum relative to healthy controls. Circulating biomarkers—including proteins, nucleic acids, and small molecules—are released into the bloodstream during disease progression and serve as indicators for early diagnosis and disease monitoring [128]. For sepsis diagnosis, a multiplexed SERS sensor targeting procalcitonin (PCT), interleukin-6 (IL-6), and C-reactive protein (CRP) achieved LODs of 0.05 ng mL^−1^, 0.02 pg mL^−1^, and 0.1 μg mL^−1^, respectively. Average PCT and IL-6 levels in sepsis patients were 5.2 ± 1.8 ng mL^−1^ and 45.3 ± 1.8 pg mL^−1^, respectively, compared with healthy controls [129]. Detection of tumor-specific circulating tumor DNA (ctDNA) has further enabled mutation identification at concentrations as low as 1 copy mL^−1^, providing faster, on-site early cancer diagnosis than conventional liquid biopsy methods [130].

These quantitative SERS detection platforms can be directly integrated into point-of-care settings such as emergency rooms and clinical screening centers, supporting real-time decision-making in emergency diagnosis, infectious disease prediction, and cancer monitoring. Pathogens, exosomes, and circulating biomarkers are prone to signal interference in complex biological matrices, but the multi-analytical capabilities and high chemical selectivity offered by SERS enhance clinical accessibility. Nevertheless, in actual patient samples, broad concentration distributions and structural heterogeneity make signal stability more sensitive to sample quality than to instrument performance. These characteristics simultaneously highlight the strengths of SERS-based point-of-care diagnostics and the challenges of standardization that remain to be addressed.

### Environmental and food safety analytes: small molecules and toxins

In environmental contamination and food safety applications, target substances typically include small molecules and protein toxins such as pesticides, heavy metals, microbial toxins, and endocrine-disrupting chemicals (EDCs). SERS biosensors provide high specificity and sensitivity for these analytes, employing molecularly imprinted, antibody-based, or enzyme-based sensing strategies that recognize distinct molecular structures [131, 132].

For pesticide residue detection, a gold nanoparticle (AuNP)-based SERS sensor quantified chlorpyrifos, an organophosphate insecticide, with an LOD of 0.5 μg L^−1^ and a quantitative range of 1–100 μg L^−1^, confirming residual levels below 8.3 μg kg^−1^ in real apple samples [133]. For heavy metal monitoring, a molecularly imprinted nanostructure-based SERS platform simultaneously quantified As, Cd, and Pb, achieving LODs of 0.1, 0.05, and 0.2 μg L^−1^, respectively, and detecting contamination levels of 1 μg L^−1^ for As, 0.8 ± 0.2 μg L^−1^ for Cd, and 3.2 ± 0.6 μg L^−1^ for Pb, thereby assessing compliance with drinking water standards [134].

For mycotoxin detection, particularly aflatoxin B_1_, a SERS sensor incorporating an isotope-labeled internal standard achieved an LOD of 0.1 ng mL^−1^ with a linear dynamic range of 0.5–50 ng mL^−1^, quantifying corn samples at 3 ng g^−1^ and verifying their suitability for distribution against the regulatory limit of 4 ng g^−1^ [135]. For the quantification of EDCs, such as bisphenol A (BPA), a silver nanorod (AgNR)-based SERS platform achieved an LOD of 5 ng L^−1^ and a quantitative range of 10–500 ng L^−1^, enabling real-time monitoring of BPA levels in drinking water and plastic container leachates [136].

These quantitative SERS-based analyses are applied in field monitoring, regulatory compliance testing, and rapid response to contamination events, thereby safeguarding public and ecological health. The performance characteristics of SERS biosensors for clinically and environmentally relevant analytes are summarized in Table 2, which provides representative detection limits, dynamic ranges, and unique features for each application category, highlighting both recent advancements and remaining limitations of quantitative SERS-based platforms.

**Table 2:** Quantitative performance of SERS biosensors for nucleic acids, proteins, pathogens, and environmental analytes

Application	Target	Material / Platform	LOD	Linear range	Result	Key significance	References
Nucleic acids / Proteins	DNA / miRNA	DNA@MNR1-21	1 fM	10 fM–10 pM	6 orders of magnitude	Isotope-labeled internal standard; 8.5-fold higher sensitivity in patients	[125]
	PSA	Au nanoparticle sensor	0.5 ng mL^−1^	1–100 ng mL^−1^	Clinical cutoff at 4 ng mL^−1^	Gold nanoparticle sensor; prostate cancer screening	[123]
	Aβ42	Sandwich immunoassay	10 pg mL^−1^	50 pg mL^−1^–10 ng mL^−1^	Patient vs. control discrimination	Alzheimer’s biomarker analysis	[124]
	SNP	SNP detection platform	~ 3 nM	Single-nucleotide resolution	Sequence-specific discrimination	Genotyping-capable SERS analysis	[120]
Pathogens / Biomarkers	SARS-CoV-2	Immunosensor	0.08 pg mL^−1^	0.1–100 pg mL^−1^	> 95% sensitivity and specificity	Rapid, point-of-care applicability	[127]
	Exosome	SERS platform	50 exosomes μL^−1^	50–500,000 exosomes μL^−1^	5 orders of magnitude	12-fold higher level in patient serum	[128]
	PCT / 6-CRP	Multiplex immunoassay	0.05 ng mL^−1^ / 0.1 ng mL^−1^	Practical detection limits: 5.2 ng mL^−1^ / 45.3 ng mL^−1^	Patient vs. control discrimination	Multiplexed sepsis biomarker analysis	[129]
	ctDNA	Liquid biopsy platform	1 copy mL^−1^	Ultra-low level detection	Tumor mutation identification	Rapid liquid biopsy applicability	[130]
Environment / Food safety	Chlorpyrifos	AuNP-based SERS sensor	0.5 μg L^−1^	1–100 μg L^−1^	< 8.3 μg kg^−1^ in apple	Pesticide residue monitoring	[133]
	As / Cd / Pb	Molecularly imprinted SERS platform	0.1 / 0.05 / 0.2 μg L^−1^	–	Water: As 1.0 μg L^−1^, Cd 0.8 ± 0.2 μg L^−1^, Pb 3.2 ± 0.6 μg L^−1^	Multiplex heavy metal monitoring	[134]
	Aflatoxin B1	Isotope-labeled SERS sensor	0.1 ng mL^−1^	0.5–50 ng mL^−1^	3 ng g^−1^ in corn	Regulatory-limit comparison	[135]
	Bisphenol A	AgNR-based SERS platform	5 ng L^−1^	10–500 ng L^−1^	Real-time monitoring in water and leachate	Endocrine disruptor monitoring	[136]

In environmental and food analysis, the chemical diversity of targets and the complexity of sample matrices (matrix effects) significantly impact quantitative reliability. For small-molecule toxicants in particular, surface adsorption characteristics and substrate selectivity often represent more critical limiting factors than quantitative accuracy itself. From this perspective, SERS requires not only high-sensitivity measurement capabilities but also structural designs capable of interpreting signals within diverse chemical backgrounds.

## Challenges, standards, and future directions

### Reproducibility, reference materials, and inter-laboratory comparability

Reproducibility—defined as the ability to obtain consistent outcomes when experiments are repeated under identical conditions and protocols—is a critical requirement for quantitative SERS biosensors. It is a prerequisite for reliable clinical diagnostics, environmental monitoring, and large-scale commercialization, necessitating standardized reference materials, comparison samples, and internationally harmonized measurement protocols [137, 138].

However, in practice, platform-specific nanostructure fabrication processes, batch-to-batch variations, sample purity, and differences in analytical environments across laboratories often lead to substantial discrepancies in numerical results, even when identical samples are analyzed. Although institutions such as the National Institute of Standards and Technology (NIST) provide reference materials for SERS, these are generally limited to simple compositions (e.g., single organic molecules) and are therefore unsuitable for complex biological or clinical matrices. This lack of inter-laboratory comparability remains a major obstacle to global standardization, regulatory approval, and clinical translation [139, 140].

These challenges, repeatedly noted by both academic and industrial communities, represent fundamental “structural weaknesses” of surface-enhanced Raman spectroscopy: signal deviation, protocol heterogeneity, and insufficient quality assurance and quality control (QA/QC). From a critical standpoint, the current research framework alone is insufficient to overcome these intrinsic limitations [141, 142].

Therefore, the development of new standardized reference materials, automated data processing systems, and objective QC guidelines that can be consistently applied across diverse experimental settings is urgently required. Looking forward, the clinical and industrial realization of quantitative SERS biosensors will depend on the establishment of international standardization networks, collaborative round-robin studies to assess the reliability of quantitative benchmarks, and the adoption of “absolute standard” signal-matching strategies across different platforms.

### Device integration, throughput, and manufacturing for translation

The practical implementation of SERS biosensors faces several critical challenges, including the mechanical and electronic integration of sensor components, scalable manufacturing throughput, and standardized production protocols. Miniaturization, automation, and integrated packaging promise improved speed and user convenience for clinical and field diagnostics, yet progress remains constrained by the microheterogeneity of nanostructured surfaces, the durability of polymer and microfluidic substrates, and their susceptibility to environmental fluctuations [12, 143].

From a production standpoint, large-scale fabrication of SERS-active substrates still faces important challenges related to lot-to-lot variation, signal consistency, and process validation. At the same time, the manufacturing landscape is evolving beyond small-batch laboratory preparation. Approaches such as nanoimprint-based nanopattern replication, roll-to-roll processing, template-assisted large-area assembly, and emerging additive manufacturing methods have begun to demonstrate improved throughput and more reproducible substrate formation, indicating that scalable production is becoming increasingly feasible for selected platform designs [144, 145].

Even so, manufacturing readiness should be evaluated not only by output volume but also by retention of analytical performance after scale-up. In this context, the most promising translational strategies are those that combine scalable fabrication with in-line quality monitoring, standardized surface functionalization, and robust packaging protocols. Thus, rather than viewing manufacturability as a binary limitation, current evidence suggests that SERS production is moving toward a more realistic intermediate stage in which scalable fabrication is achievable, but successful translation will depend on whether reproducibility and QA/QC control can be maintained across the full production workflow [146].

A critical evaluation of these technologies highlights the urgent need for innovative materials capable of resolving trade-offs among nanostructural functionality, production scalability, processing throughput, and automated analytical performance. Real-time monitoring of fabrication quality and standardized packaging optimized for integrated diagnostic systems will also be essential. In addition, the regulatory translation of SERS based diagnostics will require validated analytical performance, lot to lot consistency, defined quality control criteria, and standardized clinical testing workflows that align with broader in vitro diagnostic expectations. Without such regulatory and standardization frameworks, SERS biosensors are unlikely to advance beyond proof-of-concept demonstrations and achieve true clinical and industrial translation [147, 148].

### Emerging themes: single-molecule quantitation, in vivo SERS, and sustainable materials

Several studies have demonstrated that single molecule SERS can be achieved in highly controlled analytical systems. For example, digital SERS platforms have used event counting in confined or flow based formats to detect extremely small numbers of analyte molecules, while some bioassay formats have combined signal amplification and nanoparticle labeling to reach near single molecule sensitivity [149]. These examples support the feasibility of single molecule detection in carefully engineered platforms, particularly when the target concentration is very low and the measurement environment is tightly controlled.

However, these implementations also show clear limitations. Reliable single molecule detection usually depends on precise hotspot formation, very low analyte occupancy, specialized substrate design, and rigorous statistical interpretation of rare events. Because of these requirements, reproducibility, throughput, and applicability to complex real samples remain limited. Therefore, single molecule SERS should be considered a promising but still highly specialized approach rather than a broadly applicable solution for routine quantitative analysis [150].

In vivo SERS seeks to extend the technology beyond controlled laboratory environments into complex biological systems. To mitigate challenges such as nonspecific surface adsorption, poor biodegradability, and long-term toxicity, biocompatible coatings, targeting ligands, micro/nano-insertion devices for rapid signal acquisition, and real-time bioenvironmental monitoring algorithms have been introduced [151]. These approaches have opened access to biological phenomena previously inaccessible by conventional techniques, including molecular dynamics within tissues, drug delivery processes, and cancer microenvironment profiling. Notably, a SERS microneedle biosensor reported in 2024 demonstrated real-time enzyme monitoring in skin tissue via a minimally invasive platform [152].

The field of sustainable materials focuses on addressing the structural and environmental drawbacks of noble metal–based SERS substrates, such as high cost, resource scarcity, and ecological impact during large-scale production [153]. Recent progress includes the development of biodegradable polymers, carbon-based composite materials, and environmentally benign fabrication processes, which reduce sensor waste and enable material recycling. These systems have demonstrated both practicality and sustainability while maintaining detection yields down to 10^−9^ M [154–156].

Future development of SERS will require more than just improvements in the performance of individual materials. Two-dimensional materials and quantum dots can provide functional extensibility in terms of signal modulation, multiplexing, and chemical enhancement. Microfluidics can precisely control sample handling and reaction conditions, increasing reproducibility and throughput. Artificial intelligence and big data analytics can contribute to the quantitative analysis of complex spectra, disease classification, differentiation of multiple analytes, and inter-institutional data integration. Ultimately, these emerging themes will converge not on independent development, but on enhancing the practicality and translatability of SERS through the integration of material design, analytical systems, and data processing [157] (Table 3).

**Table 3:** Recent advances in single-molecule quantitation, in vivo SERS, and sustainable material-based platforms

Emerging theme	Study/Application (Year)	Limit of Detection	Key Feature	Reference
Single-molecule quantitation	Digital SERS in flow	Single-molecule level	Digital event counting; quantitative analysis	[158]
Single-molecule quantitation	Single-molecule telomerase	5 × 10^−14^ IU (~ 3 cells)	Signal amplification; quantum dot labeling	[159]
In Vivo SERS	SERS microneedle	~ nM–pM (in situ)	Real-time dermal tyrosinase monitoring	[152]
In Vivo SERS	Tumor imaging	326 fM (nanotag detection)	MRI/CT imaging, in vivo targeting	[160]
Sustainable materials	Au@Cu_2_O-Ag recyclable	1 nM (malachite green)	Photocatalytic substrate; reusable up to 6 cycles	[161]
Sustainable materials	AgNP–bacterial nanocellulose (BNC) paper SERS substrate	5 × 10^−7^ M (methomyl)	Fully biodegradable BNC paper; eco-friendly, flexible “paste-and-read” SERS substrate for in situ pesticide detection	[162]

### Integration with microfluidics, artificial intelligence, and big data analytics

The future progress of SERS will rely not only on improving substrate sensitivity, but also on more effective integration of sample preparation, spectral analysis, and data use. Microfluidics can improve measurement consistency by controlling sample flow and reaction conditions, artificial intelligence can assist in interpreting complex spectra, and big data analytics can support large scale comparison, validation, and standardization [163]. Together, these advances can improve reproducibility, analytical reliability, and practical usability, helping accelerate the clinical and environmental application of SERS [164].

## Conclusion

Plasmonic and SERS nanobiosensors represent powerful platforms for quantitative molecular detection. Electromagnetic and chemical enhancement of plasmonic resonance enables extreme signal amplification, while hotspot formation and structural uniformity ensure reproducible signal generation. Diverse nanostructure designs—including colloidal nanoparticles, nanopatterned substrates, and hybrid platforms—offer unique advantages in flexibility, capacity, and multifunctionality.

Precise surface chemistry control enables simultaneous analyte immobilization, molecular specificity, and suppression of background interference. SAMs, targeted biolabeling, and antifouling strategies establish the foundation for reliable quantification. Internal standards, ratiometric calibration, and isotope encoding correct for signal fluctuations, enabling absolute quantification. Rigorous calibration models and metrological standards define detection limits and dynamic ranges, while machine learning and multivariate regression facilitate accurate concentration prediction from complex spectral datasets.

SERS biosensors have demonstrated picomolar-level sensitivity and clinically relevant quantification for nucleic acids, proteins, pathogens, circulating biomarkers, and environmental toxins. Nevertheless, reproducibility, standardization, and large-scale manufacturing remain major challenges. Clinical translation continues to be hindered by the absence of internationally recognized reference materials, as well as by inter-laboratory variability and batch inconsistency.

Emerging directions—including single-molecule quantification, in vivo SERS, and sustainable materials—offer next-generation solutions to these limitations. The realization of fully quantitative SERS biosensors will require integrated progress in technological innovation, global standardization, and regulatory harmonization. Collectively, these advances will establish SERS as a quantitative benchmark for both clinical and field diagnostics.

## Data Availability

No datasets were generated or analysed during the current study.
